# Complete Genome Sequence of Klebsiella pneumoniae Phage Sweeny

**DOI:** 10.1128/MRA.01047-19

**Published:** 2019-09-26

**Authors:** Nicholas Martinez, Eric Williams, Heather Newkirk, Mei Liu, Jason J. Gill, Jolene Ramsey

**Affiliations:** a Center for Phage Technology, Texas A&M University, College Station, Texas, USA; Loyola University Chicago

## Abstract

Klebsiella pneumoniae is a multidrug-resistant bacterium causing many severe hospital-acquired infections. Here, we describe siphophage Sweeny that infects K. pneumoniae. Of its 78 predicted protein-encoding genes, a functional assignment was given to 36 of them. Sweeny is most closely related to T1-like phages at the protein level.

## ANNOUNCEMENT

Klebsiella pneumoniae is a multidrug-resistant bacterium. Due to the *bla*_KPC_ carbapenemase gene and strains of sequence type 258 (ST258), K. pneumoniae is a leading cause of hospital-acquired infection in North America and is an emerging pandemic threat (1, 2). Bacteriophages are being pursued as an alternative to antibiotics for K. pneumoniae treatment. Here, we describe the K. pneumoniae siphophage Sweeny.

Phage Sweeny was isolated from filtered (0.2-μm pore size) wastewater collected in College Station, TX. The host, a pKpQIL plasmid-cured derivative of K. pneumoniae strain 1776c, was grown aerobically in tryptic soy broth or agar (Difco) at 37°C, and phage propagation was done using the soft agar overlay method (3, 4). The genomic DNA was purified by the shotgun library preparation protocol modification of a Promega Wizard DNA clean-up system, prepared for sequencing with a TruSeq Nano low-throughput kit, and sequenced by an Illumina MiSeq platform with paired-end 250-bp reads (5). The phage-containing index yielded 876,587 total reads. These were quality controlled by FastQC (http://www.bioinformatics.babraham.ac.uk/projects/fastqc/) and trimmed with the FASTX-Toolkit v0.0.14 (http://hannonlab.cshl.edu/fastx_toolkit/). Finally, assembly with SPAdes v3.5.0 gave a contig sequence with 369-fold coverage (6). PCR off the contig ends (forward primer, 5′-CGGAACCGATCCGAAGATAAA-3′, and reverse primer, 5′-ATCGAGAAGGGACAGGTATGA-3′), followed by Sanger sequencing, was used to close and confirm the genome sequence. Glimmer v3.0 and MetaGeneAnnotator v1.0 were used to complete the structural annotation (7, 8). Rho-independent terminators were analyzed with TransTermHP v2.09 (9). Gene functions were predicted using domain searches with InterProScan v5.22-61 and protein similarity with BLAST v2.2.31 versus NCBI nonredundant, UniProtKB Swiss-Prot, and TrEMBL databases, with a 0.001 maximum expectation value cutoff (10–12). Additional evidence came from using HHpred (with the HHblits ummiclust30_2018_08 database for multiple sequence alignment [MSA] generation and PDB_mmCIF70 for modeling in HHsuite v3.0) and TMHMM v2.09 (13, 14). The annotation tools were used at default parameters, unless otherwise stated above, in the Galaxy and Web Apollo instances hosted by the Center for Phage Technology (https://cpt.tamu.edu/galaxy-pub) (15, 16). Sweeny was negatively stained with 2% (wt/vol) uranyl acetate and viewed by transmission electron microscopy at the Texas A&M Microscopy and Imaging Center (17).

Sweeny has a genome length of 50,241 bp, with a G+C content of 50.7%. The coding density was 92.8%, with 78 protein-coding genes predicted, but no tRNAs were identified by ARAGORN v2.36 (18). Sweeny is likely to use headful “pac”-type packaging, as predicted by PhageTerm (19). By progressiveMauve v2.4.0, Sweeny is most closely related to the Klebsiella phage Sushi (GenBank accession number KT001920), with 90% nucleotide identity and 73 similar proteins (20). Of the 36 predicted proteins with an assigned function, 35 Sweeny proteins have BLASTp hits to phage T1 proteins (NCBI RefSeq accession number NC_005833). Similar to phage T1, Sweeny does not encode a DNA polymerase, indicating that replication of the genome is reliant on the host replication enzymes. In Sweeny, the pinholin (NCBI accession number QEG07170), signal-arrest-release (SAR) endolysin (NCBI accession number QEG07171), and unimolecular spanin (GenBank accession number QEG07172) are directly related to the T1 lysis cassette (21).

### Data availability.

The genome sequence and associated data for phage Sweeny were deposited under GenBank accession number MK931443, BioProject accession number PRJNA222858, SRA accession number SRR8869234, and BioSample accession number SAMN11360432.
